# Record-level, exceptionally broadband borophene-based absorber with near-perfect absorption: design and comparison with a graphene-based counterpart

**DOI:** 10.1515/nanoph-2025-0391

**Published:** 2025-11-10

**Authors:** Amir Ali Marefati, Mahdieh Bozorgi

**Affiliations:** University of Zanjan, Zanjan, Iran

**Keywords:** absorber, broadband, infrared, borophene, graphene

## Abstract

We present a high performance ultra-broadband optical absorber based on a metal–insulator–metal (MIM) configuration, enhanced by two-dimensional (2D) materials: graphene and borophene. The base design includes a titanium resonator, an SiO_2_ dielectric spacer, and a gold ground plane. Performance optimization is achieved through integration of 2D materials, anti-reflection coatings (ARC), and tuning of structural parameters, Fermi energy, and surface carrier density. Numerical simulations using the finite difference time domain (FDTD) method show that incorporating borophene, due to its exceptionally high carrier density, leads to remarkable enhancement in both absorption amplitude and spectral bandwidth. When integrated with an optimized antireflection coating (ARC), the borophene-based absorber achieves over 90 % absorption across 790–3,232 nm (bandwidth: 2,442 nm), corresponding to a 136 % enhancement over the base design. For absorption above 80 %, the bandwidth extends from 760 to 3,306 nm (2,546 nm), yielding a 125 % improvement. The associated fractional bandwidths are 121 % and 125 %, respectively. By comparison, the graphene-based counterpart, with a properly tuned ARC and Fermi level, delivers over 90 % absorption within 923–2,108 nm (1,185 nm, 13 % improvement), while maintaining absorption above 80 % across 911–2,256 nm (1,345 nm, 12 % improvement), with corresponding fractional bandwidths of 78 % and 84 %. Comparative analysis underscores the critical importance of 2D material selection and placement, ARC and resonator optimization, and optical tuning in achieving optimal performance. These results indicate strong potential for practical applications in advanced optoelectronic and photonic devices, including infrared imaging, optical sensing, broadband photodetectors, solar energy harvesting, and stealth or thermal camouflage systems.

## 1 Introduction

In recent years, two-dimensional materials such as graphene and borophene have gained significant attention due to their remarkable properties, including high thermal conductivity, excellent mechanical and electronic characteristics, and anisotropic optical behavior [1], [2]. In particular, borophene, with its high carrier density (∼10^19^ m^−2^), several orders of magnitude higher than other two-dimensional (2D) materials such as graphene (∼10^16^ m^−2^) and black phosphorus (∼10^17^ m^−2^), holds great potential for applications in plasmonic devices and refractive index sensing [1], [3], [4]. This feature enables borophene-based devices to work effectively in the visible and near-infrared regions [1]. Metamaterials, with their ability to design nanostructures that enable broad-band absorption and dynamic control of optical properties, also hold vast potential for various applications. The combination of these materials with graphene and borophene can significantly enhance sensor performance and optical absorption characteristics. In addition to their unique intrinsic properties, two-dimensional materials like borophene and graphene also show great promise as tunable absorbers [5]. The high electron density of borophene allows it to support plasmonic resonances in the near-infrared region. By applying external stimuli such as voltage or chemical doping, it is possible to dynamically modify the electron density of borophene, resulting in a change in its conductivity [1], [3], [6]. Similarly, the surface conductivity of graphene, an important optoelectronic property, can be controlled by adjusting the chemical potential through external gate voltages, magnetic fields, or optical excitation. These tunable properties make both materials highly promising candidates for dynamic metamaterial systems, especially in the near-infrared region [7].

Graphene effectively enhances absorption and tunability. For example, in reference [7], a monolayer graphene on a metal film significantly increased near-infrared absorption, raising peak values from 0.67 and 0.88 to 0.98 and 0.91, respectively. Cao et al. developed a tunable graphene absorber based on asymmetric gratings, enabling absorption modulation from 61.73 % up to 99.99 % in the near-infrared by varying the Fermi energy and grating parameters [8]. Additionally, reference [5] reports a broadband plasmonic metamaterial absorber combining graphene with high melting point lossy metals like Ni, Ti, Mn, and W. This design not only improves absorption efficiency by over 10 % but also provides surface protection and thermal stability, achieving broad absorption from 400 nm to 2,200 nm with over 95 % average absorption between 700 nm and 2,000 nm. These results emphasize graphene’s key role in boosting absorption and its potential for tunable metamaterial applications. For instance, Tang et al. achieved ultra-broadband near-infrared absorption enhancement of monolayer graphene by employing multiple magnetic plasmon resonators on nanostructured metal surfaces, resulting in over 60 % absorption from 850 nm to 1,500 nm [9]. Patel et al. proposed a graphene-based broadband solar absorber by sandwiching graphene between dielectric and resonator layers, improving absorption across a wide spectrum including visible, infrared, and ultraviolet [10]. Pourhossein Bagheri et al. designed and optimized a graphene absorber with metal films patterned by annular and L-shaped grooves, achieving an average absorption rate of 85.79 % in the visible to near-infrared range [11].

While graphene has been extensively explored for tunable optical absorption through electrostatic gating, recent studies reveal that borophene offers comparable and in some cases superior tunability, especially in the near-infrared and terahertz regimes. In ref. [1], a borophene-based absorber with three absorption peaks has been designed. By changing the carrier density, both the wavelength and absorption amplitude of the absorber can be tuned. Also, in reference [4], by adjusting the electron density, both the absorption peak and absorption intensity change dynamically in addition to these, Almawgani et al. numerically demonstrated a multilayer borophene-based absorber with over 90 % peak absorption across UV and IR ranges, stable under varying incident angles [12]. Alharbi et al. proposed a borophene–silica–silver layered refractive index sensor operating in the infrared spectrum with high sensitivity and wide-angle stability [3]. Liu et al. designed a borophene absorber achieving 99.8 % absorption via critical coupling in the visible range with strong anisotropic and polarization-dependent properties [13]. Lin et al. introduced a borophene-based anisotropic metamaterial perfect absorber exhibiting triple-band absorption and tunable resonances for biochemical sensing [4]. Finally, Liu et al. presented a VO_2_-integrated borophene broadband terahertz absorber with dynamically tunable absorption peaks and excellent angle tolerance [2].

The anti-reflection coating (ARC) plays a crucial role in enhancing the absorption performance of metamaterial absorbers. In ref. [14], when SiO_2_ ARC is added to the structure, the 90 % absorption bandwidth significantly increases from 747 nm (836–1,583 nm) to 1,684 nm (440–2,124 nm), and the average absorption increases from 95.9 % to 96.2 %. The spectrum also reveals multiple absorption peaks, indicating the excitation of multiple resonant modes, which is beneficial for constructing ultra-broadband absorbers. Additionally, when the ARC material is changed to Si_3_N_4_, the absorber’s performance in the longer wavelength range improves even more. In this case, the absorber achieves an average absorption rate of 95.0 % over a wavelength range of 833–2,994 nm. These results highlight the effectiveness of ARC in improving both the bandwidth and the flexibility of the operating band of the absorber.

In this study, we propose a broadband metamaterial absorber based on a metal–insulator–metal (MIM) configuration, incorporating two-dimensional materials – graphene and borophene – to optimize and compare optical performance. To further enhance absorption efficiency, anti-reflection coatings made of SiO_2_ and Si_3_N_4_ are employed. The absorber structure consists of a titanium resonator, a SiO_2_ dielectric spacer, and a gold ground plane. This work presents the first design and numerical demonstration of an ultra-broadband borophene-based absorber that significantly outperforms its graphene-based counterpart in both absorption strength and spectral bandwidth. The integration of borophene, owing to its exceptionally high carrier density, leads to substantial improvements in both amplitude and bandwidth of absorption. Numerical results show that the proposed structure achieves over 90 %, 80 % absorption across a bandwidth of 2,442 nm, 2,546 nm corresponding to a 136 % and 125 % improvement compared to the base structure and the graphene-based design, respectively. These findings underscore the great potential of borophene-based absorbers for next-generation applications in solar energy harvesting, thermal energy conversion, infrared sensing, and surface disinfection technologies.

## 2 Design, materials, and methods

### 2.1 Design

**Figure 1:**
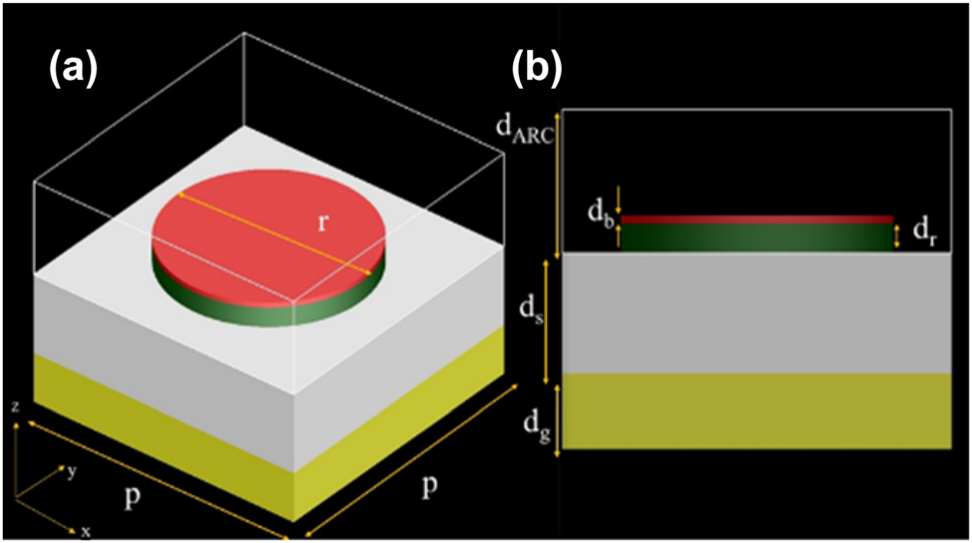
Schematic representation of the proposed broadband absorber structure based on 2D materials. (a) Top view of the designed broadband absorber structure, where the period of the unit cell is *p* = 600 nm and the diameter of the circular resonator is *r* = 420 nm. (b) Side view of the multilayer absorber showing the thicknesses of each component: the ground gold layer (*d*_*g*_ = 100 nm), the dielectric spacer (*d*_*s*_ = 160 nm), the ARC layer (*d*_ARC_ = 190 nm), the circular resonator (*d*_*r*_ = 30 nm), and the top 2D material layer (graphene or borophene) (*d*_*b*_ = 0.3 nm).

In Figure 1, the design of the broadband absorber structure is shown. Structure with p period consists of several layers arranged in the following order from bottom to top:

1. Reflective gold (Au [15]) layer with thickness *d*_*g*_.
2. Dielectric layer of silicon dioxide (SiO_2_ [16]) with thickness *d*_*s*_.
3. Resonator layer of titanium (Ti [16]) with thickness *d*_*r*_ and diameter *r*.
4. Circular borophene layer with thickness *d*_*b*_: which imparts specific optical properties to the device.
5. ARC layer with thickness *d*_ARC_: which improves light absorption and reduces reflection across the spectrum.

### 2.2 Materials

#### 2.2.1 ARC layer

The selection of a suitable material for the anti-reflection coating layer plays a key role in enhancing the performance of the broadband optical absorbers. This selection is guided by three main criteria:

1. transparency or translucency: To achieve effective absorption across a wide spectral range from visible to infrared, the ARC material must be transparent or translucent in this range. This helps reduce surface reflection and allows more light to enter the structure.
2. compatibility with plasmonic excitation conditions: The optical properties of the ARC material must support the conditions necessary for exciting surface or localized plasmons. In this design, two ARC materials are used: silicon dioxide (SiO_2_) and silicon nitride (Si_3_N_4_), each with distinct advantages:
  – SiO_2_, with its low refractive index, effectively reduces reflection in the IR band and enhances light transmission to underlying layers.
  – Si_3_N_4_, with a higher refractive index, improves impedance matching between the surrounding medium and the resonator, optimizing absorption at specific frequency bands.
3. ease of fabrication and implementation: The ARC material should be easily fabricated in both laboratory and industrial settings to enable large-scale and cost-effective production.

This thoughtful selection allows the structure to minimize optical reflection while simultaneously supporting resonant optical modes, thereby improving overall light absorption efficiency [14].

#### 2.2.2 Borophene

As a recent addition to the family of 2D monoelemental materials, borophene has attracted considerable attention due to its outstanding thermal conductivity, excellent mechanical and electronic transport properties, and anisotropic optical behavior. One of its most significant advantages is its exceptionally high carrier density, exceeding 10^19^ m^−2^ [1], [17], [18], which is several orders of magnitude greater than that of other 2D materials such as graphene (∼10^16^ − 10^17^ m^−2^) [19], [20], [21] and black phosphorus (∼10^17^ m^−2^). This high carrier density enables borophene to operate efficiently in both the visible and near-infrared spectral ranges. Moreover, by applying an external electric bias or through chemical doping, the carrier density of borophene can be dynamically tuned, allowing real-time control over its optical conductivity. This tunability makes borophene highly suitable for use in reconfigurable and tunable optical absorbers capable of adapting to different wavelength ranges [1], [3], [4], [6], [13], [22], [23], [24], [25]. In addition, borophene, like other 2D materials, provides strong light confinement at the atomic scale due to its ultra-thin nature and large surface-to-volume ratio. This results in enhanced light–matter interaction, leading to increased sensitivity in sensing applications and improved optical performance in plasmonic devices [13], [26]. The conductivity of borophene is calculated using the Drude model:

(1) $$\sigma_{jj}=\frac{iD_{j}}{\pi \left(\omega +i\tau^{-1}\right)},\;D_{j}=\frac{\pi e^{2}n_{s}}{m_{j}}$$

In Equation (1), *j* refers to the crystallographic direction of borophene (either *x* or *y*), highlighting the material’s anisotropic nature and *i* is the imaginary unit. The angular frequency of the incident electromagnetic wave is denoted by *ω*, and the electron relaxation time *τ* is set to 65 fs [1], [4], [18]. The Drude weight *D*_*j*_ depends on the elementary charge *e*, the surface carrier density *n*_*s*_ (assumed as 1 × 10^19^ m^−2^), and the direction-dependent effective electron mass *m*_*j*_. For borophene, these effective masses are considered to be *m*_*x*_ = 1.4 *m*_0_ and *m*_*y*_ = 5.2 *m*_0_, where *m*_0_ is the electron rest mass. These parameters describe the anisotropic optical response of borophene and are essential for accurately modeling of its conductivity. Additionally, the permittivity of borophene is calculated as:

(2) $$\varepsilon_{\mathrm{jj}}=\varepsilon_{r}+\frac{i\sigma_{\mathrm{jj}}}{\varepsilon_{0}\omega d_{b}}$$

In Equation (2) *j* refers to the crystallographic direction, *ε*_*r*_ = 11 [4], [18], [27] represents the relative permittivity of borophene, *ε*_0_ is the vacuum permittivity, and *d*_*b*_ is the thickness of the borophene monolayer. Similar to other 2D materials, the permittivity in the out-of-plane (*z*) direction is assumed to be equal to the in-plane relative permittivity *ε*_*zz*_ = *ε*_*r*_ [1], [2], [6], [13], [18].

#### 2.2.3 Graphene

Graphene is a two-dimensional material with exceptional optical and electrical properties, composed of a single atomic layer of carbon. It is particularly useful in the infrared (IR) range due to its strong optical absorption capabilities and the ability to tune its electrical conductivity through external gate voltages. Graphene is widely used in the design of optical absorbers and sensors, as well as in solar cells. Due to its ability to absorb light across a wide frequency range, including the infrared, graphene can be utilized to improve the absorption efficiency in structures that require effective light confinement and absorption, such as sensors, solar cells, and photonic devices. Graphene’s interaction with electromagnetic waves can be modeled by considering it as an ultra-thin dielectric layer. Its optical response is defined by complex surface conductivity, which includes both intraband and interband components. These components are calculated using the following expressions [5], [9], [28], [29], [30], [31]:

(3) $$\sigma_{\text{intra}}=\frac{ie^{2}K_{B}T}{\pi \hbar^{2}\left(\omega +\frac{i}{\tau }\right)}\left(\frac{E_{f}}{K_{B}T}+2\ln\left(e^{-\frac{E_{f}}{K_{B}T}}+1\right)\right)$$

(4) $$\sigma_{\text{inter}}=\frac{ie^{2}}{4\pi \hbar }\ln\left(\frac{2E_{f}-\left(\omega +\frac{i}{\tau }\right)\hbar }{2E_{f}+\left(\omega +\frac{i}{\tau }\right)\hbar }\right)$$

(5) $$\sigma =\sigma_{\text{intra}}+\sigma_{\text{inter}}$$

In these expressions, *e* is the electron charge, *K*_*B*_ is the Boltzmann constant, *T* denotes the absolute temperature, *ℏ* is the reduced Planck constant, *ω* is the angular frequency of the incident electromagnetic wave, *E*_*f*_ (∼0.1–0.9 eV) is the Fermi energy, and *τ* represents carrier scattering time which is set to 100 fs [20], [32], [33].

The anisotropic relative permittivity of graphene can be expressed as:

(6) $$\varepsilon_{\text{graphene}}=\left(\begin{array}{ccc} 1+\frac{i\sigma }{\left(\omega \varepsilon_{0}t_{g}\right)} & 0 & 0\\ 0 & 1+\frac{i\sigma }{\left(\omega \varepsilon_{0}t_{g}\right)} & 0\\ 0 & 0 & 1 \end{array}\right)$$

where *ε*_0_ is the vacuum permittivity, *t*_*g*_ is the thickness of graphene, which is assumed to be 0.3 nm in the simulations [5], [7], [8], [10], [34].

### 2.3 Methods

The interaction of light with the absorber structure is analyzed using the finite-difference time-domain (FDTD) method. A three-dimensional model of the absorber is constructed with periodic boundary conditions applied along the *x* and *y* directions to represent the periodic nature of the structure, while a perfect matched layer (PML) is used in the *z* direction to prevent reflection from the simulation boundaries. A plane wave is normally incident on the structure with the electric field polarized along the *x*-axis. Two power monitors are positioned above and below the absorber to measure the reflectance (*R*) and transmittance (*T*), respectively. The spectral absorptance (*A*) is then calculated using the following expression [30]:

(7) A=1−R−T

## 3 Results and discussion

In this section, the optical performance of the proposed absorber structure is examined and analyzed under different configurations. The absorption characteristics are evaluated based on the FDTD simulation results, considering various combinations of structural components, including the base structure, the addition of the borophene layer, the ARC, and the complete hybrid configuration.

**Figure 2:**
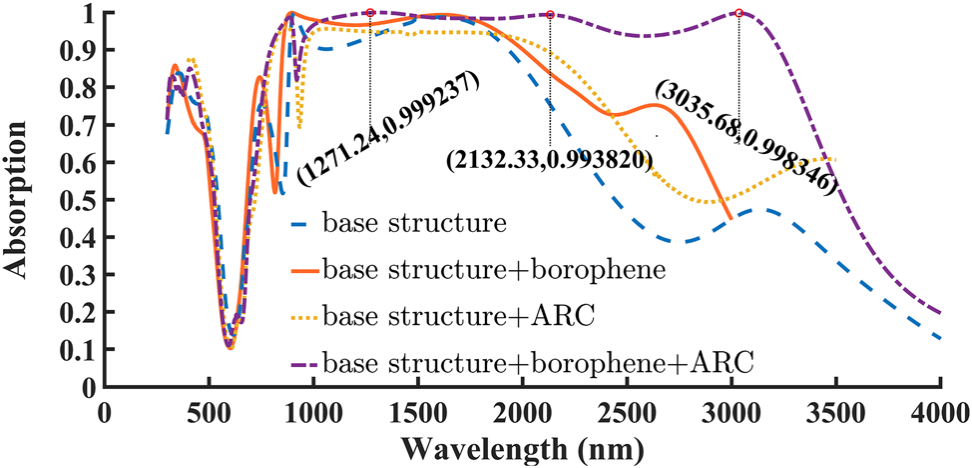
Absorption performance of the proposed absorber structure under different configurations. The blue dashed line represents the base structure, the yellow dotted line corresponds to the structure with the ARC (*d*_ARC_ = 190 nm), the orange solid line shows the performance with the borophene layer with *n*_*s*_ = 1, and the purple dash-dotted line illustrates the hybrid configuration with both borophene and ARC.

By incorporating the borophene layer into the structure, not only does the absorption amplitude increase, but the absorption bandwidth also significantly improves. In the initial structure, the bandwidth corresponding to absorption above 90 % is 1,044 nm, and for absorption above 80 %, it is 1,192 nm. After adding the borophene, these values increase to 1,147 nm (90 %) and 1,369 nm (80 %), corresponding to approximately 10 % and 15 % improvement, respectively. When only the ARC layer is introduced, the absorption amplitude slightly decreases, but the bandwidth increases. Remarkably, when both the borophene and ARC layers are simultaneously integrated, the absorber performance improves dramatically. The 90 % absorption bandwidth expands to 2,442 nm, representing about a 136 % increase, while the 80 % absorption bandwidth reaches 2,546 nm, showing approximately a 125 % enhancement. Additionally, the absorption amplitude exceeds 95 % across most of the operating wavelengths, clearly demonstrating the high efficiency of the proposed absorber structure in light confinement and absorption. These improvements are clearly illustrated in Figure 2, which visually demonstrates the enhanced absorption performance across different configurations.

**Figure 3:**
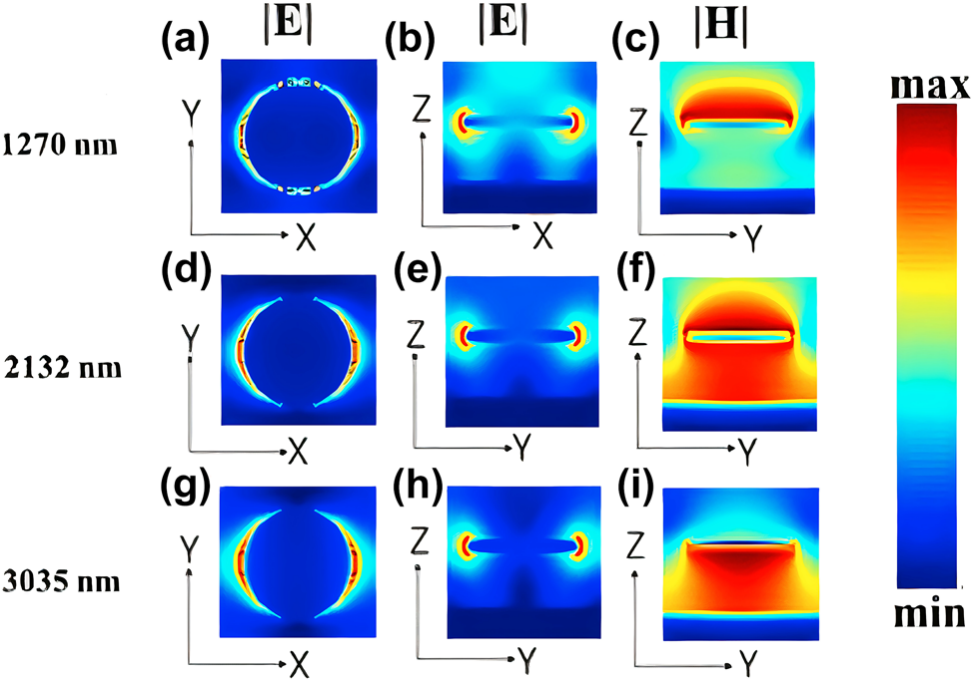
Distribution of the electric and the magnetic fields in the absorber unit cell at three main absorption wavelengths: 1,270, 2,132, and 3,035 nm, corresponding to each row. The first column shows the electric field in the *X*–*Y* plane, the second column shows the electric field in the *X*–*Z* plane, and the third column illustrates the magnetic field in the *Y*–*Z* plane.

The broadband enhancement in the borophene based structure arises from two synergistic effects: **1-**Borophene plasmonic response: Borophene’s exceptionally high surface carrier density enables strong free-carrier oscillations that couple with localized surface plasmons at the Ti–borophene interface. These hybrid plasmonic-Fabry–Pérot modes amplify the absorption amplitude across a broad spectral range. However, due to borophene’s inherently high carrier concentration, its plasmon frequency lies in the near-infrared region and is less sensitive to Fermi-level variations, leading to minimal spectral change. **2-**ARC layer function: The anti-reflection coating primarily minimizes surface reflection and improves impedance matching, thereby extending the absorption spectrum, particularly beyond 2 μm. Together, these effects yield the reported ultrabroadband absorption [1], [35]. To gain deeper insight into the absorption mechanisms at the resonant wavelengths, the spatial distributions of the electric and magnetic fields were analyzed at three absorption peaks: 1,270 nm, 2,132 nm, and 3,035 nm. These field distributions, shown in Figure 3, are presented in three orthogonal planes: *X*–*Y*, *X*–*Z*, and *Y*–*Z*. The first column depicts the electric field in the *X*–*Y* plane, the second column shows the electric field in the *X*–*Z* plane, and the third column illustrates the magnetic field in the *Y*–*Z* plane. In these figures the normal *x*-polarized light was used. The electric field maps clearly demonstrate dipolar excitation with maximum field intensities concentrated at the edges of the resonator, confirming the excitation of localized surface plasmon (LSP) modes. The magnetic field distributions further elucidate the light–structure coupling. In Figure 3c, a dominant LSP resonance is observed, evidenced by strong magnetic field localization on the resonator surface. A similar response is observed in Figure 3f, reinforcing the role of (LSPs) and their coupling with the surface plasmon polariton resonance (SPPR) between the ground metal and the circular resonator in enhancing the absorption. Moreover, Figure 3f and i reveal pronounced magnetic field confinement within the silica dielectric layer. In these cases, the incident light penetrates the thin Ti resonator and is reflected by the underlying gold layer, producing backward reflection toward the Ti surface. This interaction between the top Ti and bottom Au layers, separated by the silica spacer, gives rise to a Fabry–Pérot (FP) cavity. The penetration depth of electromagnetic waves in Ti increases with wavelength. At 1,270 nm, it is smaller than the Ti thickness, and the incident wave cannot pass through the Ti layer, resulting solely in surface plasmon excitation. At 2,132 nm, the penetration depth becomes comparable to the Ti thickness, allowing partial transmission through Ti and leading to a hybrid response that combines plasmonic excitation with FP cavity resonance. At 3,035 nm, the penetration depth exceeds the Ti thickness, enabling stronger transmission through the Ti resonator, where absorption is mainly governed by the FP cavity, although plasmon excitation still plays a role. Given the inherently lossy nature of Ti, the FP cavity exhibits it is a relatively low quality factor, producing a broadened absorption spectrum. Consequently, the combination of thin-film interference, LSP and SPPR excitations, and FP resonance synergistically contributes to achieving ultra-broadband absorption in the proposed structure.

**Figure 4:**
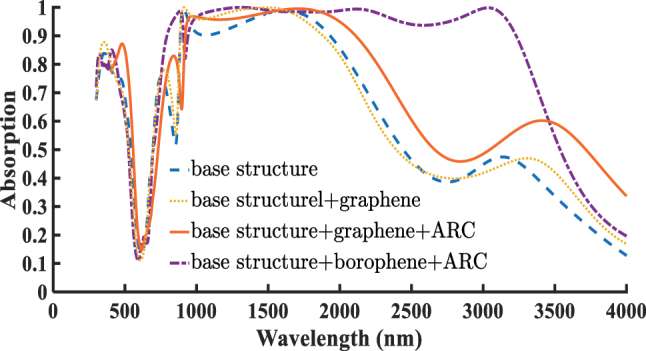
Comparison of the absorption spectra of the absorber structure under four different configurations: the base structure, the structure incorporating graphene with a Fermi energy of *E*_*f*_ = 0.9 eV, the hybrid structure with graphene and ARC layer *d*_ARC_ = 90 nm, and the aforementioned borophene-based absorber with *n*_*s*_ = 1, *d*_ARC_ = 190 nm.

In the following section, we compare the effect of incorporating graphene into the absorber structure with that of borophene. In the base structure, the absorption above 95 % spans from 1,340 to 1,838 nm, but it does not reach perfect (100 %) absorption. By incorporating the graphene layer into the structure, this high-absorption band expands significantly to the range of 891–1,773 nm, where perfect absorption is achieved at certain wavelengths. However, for the absorption above 80 %, a slight reduction in bandwidth is observed. The absorption bandwidth shrinks from 879–2,064 nm to 879–2,028 nm, reflecting a decrease of 36 nm, or approximately 1.7 % relative to the initial bandwidth of 1,185 nm. Despite this slight reduction, the graphene-based structure still outperforms the original unit cell in terms of absorption. Furthermore, by adding a 90 nm ARC to the graphene-based structure, both the magnitude and bandwidth of absorption are significantly enhanced. The absorption above 95 % extends from 930 to 2,004 nm, marking an approximate 29 % increase in bandwidth compared to the non-ARC case. Similarly, the absorption above 80 % widens from 879–2,064 nm to 911–2,254 nm, corresponding to a 12 % increase over the base structure. All these results including comparisons between the base structure, the graphene-enhanced design, and the borophene-enhanced design are clearly illustrated in Figure 4, which allows for straightforward visual evaluation of each structure’s performance. These results show that both borophene and graphene layers enhance the absorption efficiency and bandwidth of the designed absorber. However, the borophene-based absorber achieves exceptionally broadband and near-perfect absorption, outperforming its graphene counterpart. The absorption mechanisms of borophene and graphene are distinct due to their fundamentally different electronic structures and carrier concentrations. Borophene exhibits a metallic intraband response with carrier densities over 10^19^ m^−2^, enabling strong plasmonic activity in the near-infrared (NIR) range. These resonances lead to intense field confinement and strong coupling with the incident electromagnetic waves, thereby resulting in higher absorption. Thus, the higher absorption of borophene compared to graphene is primarily attributed to: higher intrinsic carrier density of borophene, lower ohmic losses, and metallic behavior in the NIR regime, and stronger plasmonic coupling between borophene and the MIM structure. In contrast, graphene with a significantly lower carrier density (*n*_*s*_∼10^16^−10^17^ m^−2^) supports tunable plasmons mainly in the mid-infrared (MIR) and terahertz regimes. Due to the much higher mobility and lower fermi energy of graphene over the borophene ( $\mu =e\nu_{f}^{2}\tau /E_{f}$ , $E_{f}=\nu_{f}\hbar \sqrt{\pi n_{s}}$ ) the loss values and mechanisms also differ: graphene absorption is strongly influenced by interband transitions and phonon scattering, whereas borophene shows metallic-like damping dominated by intraband scattering. Therefore, under the same platform and excitation wavelength, graphene does not exhibit pronounced plasmonic activity [21], [35], [36]. The absorption peak of this structure in the NIR regime can be attributed to several non-plasmonic mechanisms: Ohmic or resistive loss, Interference-induced absorption in the maltilayer structure and Fabry–Pérot cavity modes.

**Figure 5:**
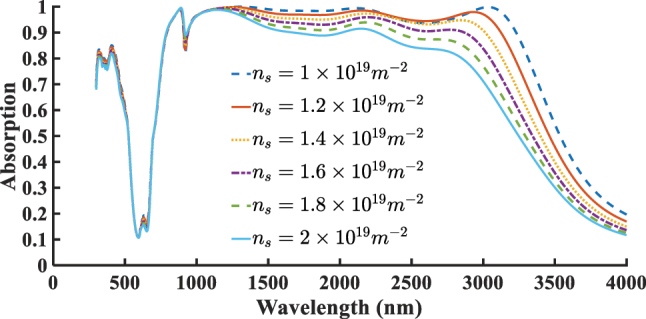
Effect of varying surface carrier density *n*_*s*_ (m^−2^) in the borophene-based structure.

**Figure 6:**
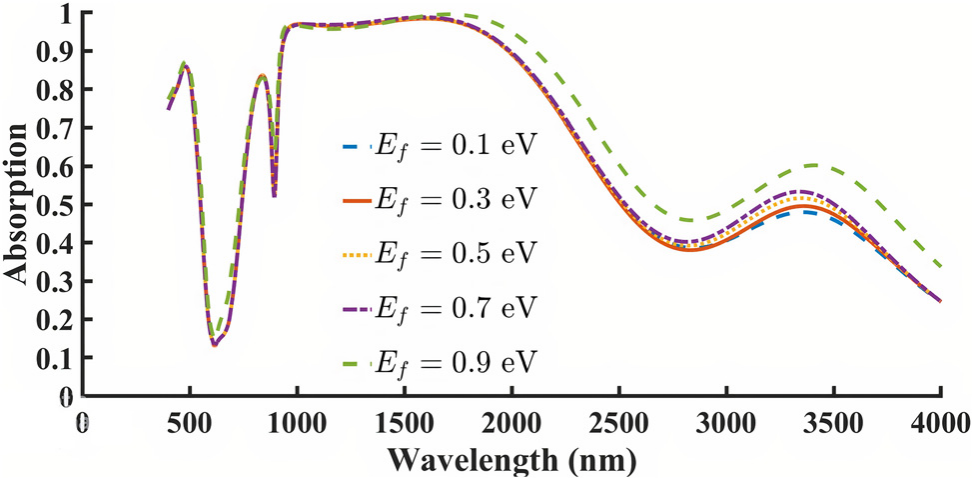
Effect of changing Fermi energy *E*_*f*_ in the graphene-based structure.

Based on both the proposed borophene- and graphene-based structures, we aim to optimize key parameters to further improve the absorber’s efficiency and broadband performance. To this end, we investigate the influence of two critical parameters on the absorption characteristics of the designed structures: the surface carrier density (*n*_*s*_) in the borophene-based structure and the Fermi energy (*E*_*f*_) in the graphene-based structure. Figure 5 presents the absorption spectra for various values of *n*_*s*_. The carrier densities assumed for borophene are supported by both theoretical predictions and experimental findings, which indicate that borophene can naturally exhibit very high intrinsic carrier densities exceeding 10^19^ m^−2^ [1], [17], [18]. As shown, increasing the surface carrier density results in a noticeable decline in both absorption intensity and bandwidth. Therefore, the optimal absorption performance is achieved when *n*_*s*_ = 1 × 10^19^ m^−2^. Figure 6 presents the effect of varying *E*_*f*_ on the graphene-based absorber. Although changes in *E*_*f*_ do not significantly shift the resonant wavelength, both the absorption magnitude and bandwidth slightly improve as *E*_*f*_ increases. The highest performance is observed at *E*_*f*_ = 0.9 eV. Achieving a Fermi level shift of 0.9 eV in graphene purely via electrostatic gating is experimentally difficult. However, this value has been reported in literature using high-*κ* dielectrics and/or dual-gate or ionic-gating architectures [37], [38], [39]. We included this upper range to explore the maximum possible tunability of the absorber. Regarding the limited tunability below 0.9 eV, this originates from the intrinsic intraband–interband transition thresholds of graphene. At low doping levels, interband transitions dominate, resulting in weak change in the absorption spectra. Only beyond a certain doping threshold does intraband plasmonic response become significant, enabling noticeable modulation of the absorption spectrum.

In this study, the tunability of graphene is expressed in terms of its Fermi energy (*E*_*f*_) which is conventional, whereas the tunability of borophene is described by its surface carrier density (*n*_*s*_) due to its metallic nature and extremely high carrier concentration. These two parameters are related by $n_{s}=E_{f}^{2}/\pi \hbar^{2}\upsilon_{F}^{2}$ , where *υ*_*F*_ = 1 × 10^6^ m/s [21], [35], [36]. Given that borophene and graphene differ by several orders of magnitude in carrier density, operate in different wavelength regimes, and exhibit distinct loss mechanisms, the primary objective here is not to establish one as superior, but rather to evaluate the same MIM platform integrated with two representative 2D materials to explore design flexibility and potential performance enhancement.

**Figure 7:**
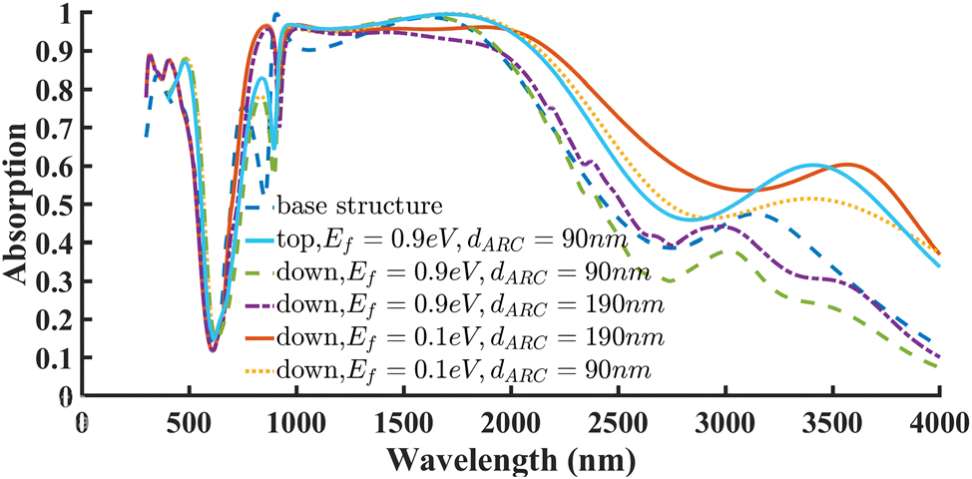
Comparison of the effect of graphene placement on top and beneath the Ti resonator with the base structure under variations in ARC thickness and Fermi energy.

**Figure 8:**
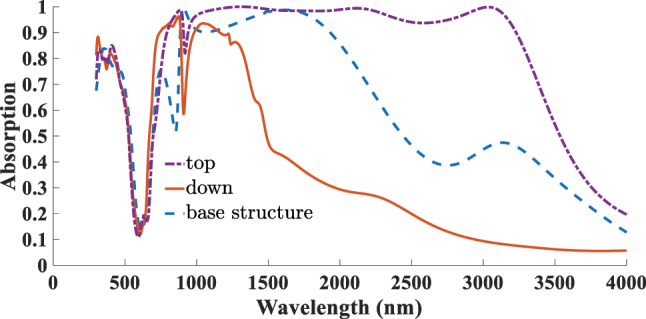
Comparison of the effect of borophene placement on top and beneath the Ti resonator with the base structure.

To further investigate the influence of 2D material placement on absorber performance, an additional configuration was considered in which the graphene and borophene layers were repositioned from the top of the resonator to the interface between the dielectric layer and the resonator. In this design, the two-dimensional sheets were modeled as square layers that fully cover the top surface of the dielectric region within the unit cell. The results of this investigation are presented in Figures 7 and 8. Figure 7 compares the absorption spectra in several cases: first circular graphene layer placed on top of the resonator at the base structure with a Fermi energy of *E*_*f*_ = 0.9, and ARC layer of 90 nm thickness, then the square graphene sheet is positioned directly beneath the Ti resonator, on top of the SiO_2_ dielectric layer. It is observed that relocating the graphene beneath the Ti resonator does not significantly alter the bandwidth and absorption. Increasing the ARC layer thickness to 190 nm results in a slight enhancement in bandwidth, although the peak absorption decreases and no longer reaches 100 %. Next, when the Fermi energy is reduced to *E*_*f*_ = 0.1, and the ARC layer thickness is set to 190 nm, a much broader and more uniform absorption band is achieved, spanning from 757 to 2,362 nm, though complete absorption (100 %) is not reached. Upon reducing the ARC thickness back to 90 nm, the absorption behavior closely resembled the case of graphene positioned at the top of the resonator, with absorption reaching 100 % at certain wavelengths. Interestingly, when graphene is placed above the resonator, increasing *E*_*f*_ improves both absorption magnitude and bandwidth. In contrast, when positioned below, reducing *E*_*f*_ leads to better absorption characteristics. This emphasizes the critical role of material placement in the performance of absorptive structures. Figure 8 shows a similar analysis for the borophene layer. Relocating the borophene beneath the Ti resonator significantly reduces both the absorption magnitude and bandwidth, resulting in a drastic deterioration of performance. These results highlight the importance of accurate positioning of 2D materials in designing high-performance absorbers.

**Figure 9:**
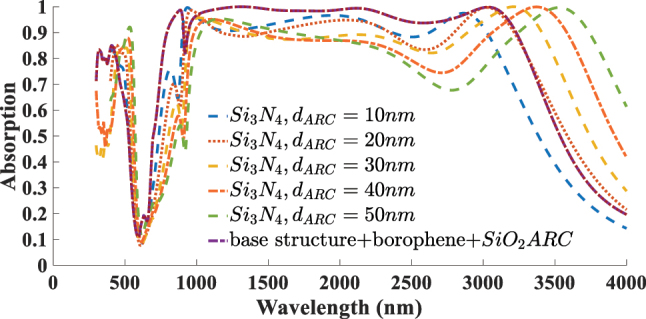
Absorption spectra of the borophene-based absorber using a Si_3_N_4_ ARC layer with varying thicknesses (10–50 nm). The results are compared to the optimal case with a 190 nm SiO_2_ ARC layer.

To enable a more detailed performance evaluation, we investigate the performance of the borophene-based absorber by replacing the conventional SiO_2_ ARC layer with a Si_3_N_4_ layer. This analysis is conducted based on the configuration that previously exhibited optimal performance featuring a borophene layer with *n*_*s*_ = 1 and an ARC thickness of 190 nm. Figure 9 presents the absorption spectra of the structure incorporating the Si_3_N_4_ layer, with its thickness varied from 10 nm to 50 nm. As observed, increasing the thickness of the ARC layer thickness causes a slight shift in the absorption bandwidth and, in some cases, leads to improved absorption efficiency. However, in certain wavelength ranges, the absorption drops below 80 %, and overall, the absorption performance does not reach the levels exceeding 95 % that were achieved with the SiO_2_-based ARC layer.

**Figure 10:**
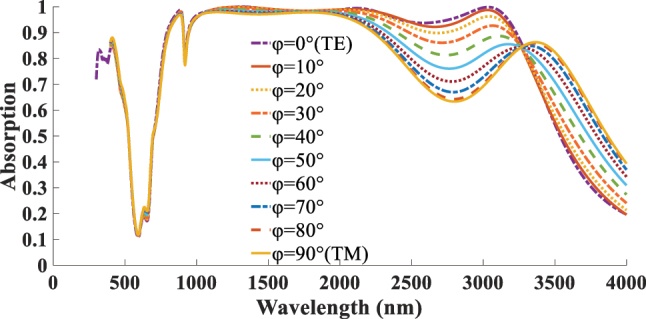
*φ* dependent absorption response of the structure under *φ* = 0 (TE) to *φ* = 90 (TM) incidents.

In the next stage of the optimized absorber design analysis, the effect of varying the polarization angle *φ* of the normal incident wave on the absorption performance is investigated. As shown in Figure 10, *φ* is varied from 0 to 90, where *φ* = 0 corresponds to TE incidence and *φ* = 90 corresponds to TM incidence. The results indicate that the maximum absorption occurs at *φ* = 0, while the absorption efficiency decreases gradually as *φ* increases toward 90. This reduction is attributed to the anisotropic response of borophene layer to different incident polarizations.

**Figure 11:**
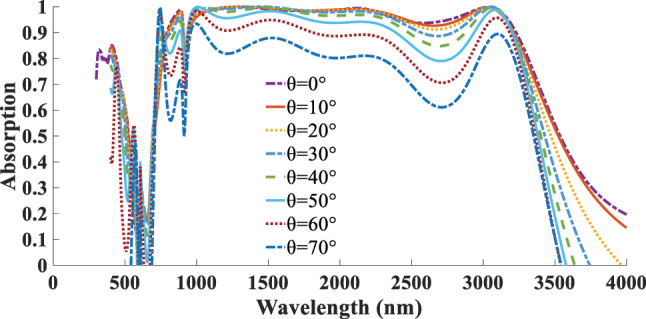
Dependent of absorption response to the *θ* incidents.

In the subsequent analysis, the absorption performance of the optimized absorber is examined as a function of the incident angle *θ*. As illustrated in Figure 11, *θ* is varied from normal incidence *θ* = 0 to oblique incidence *θ* = 70. The results demonstrate that the absorber maintains high absorption efficiency for small *θ* values, while a gradual reduction in absorption is observed as *θ* increases. This behavior is primarily attributed to the variation in the effective optical path length and impedance matching conditions with changing incidence angle.

**Figure 12:**
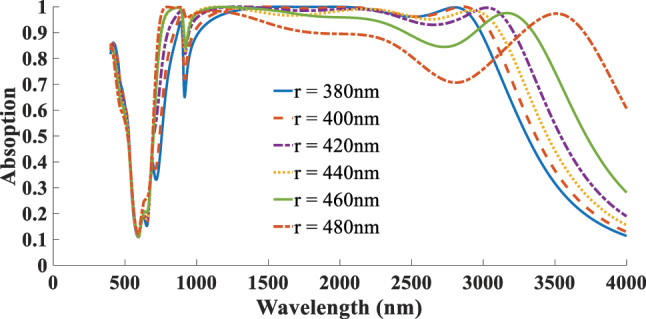
Dependent of absorption response to the resonator diameter (*r*).

**Figure 13:**
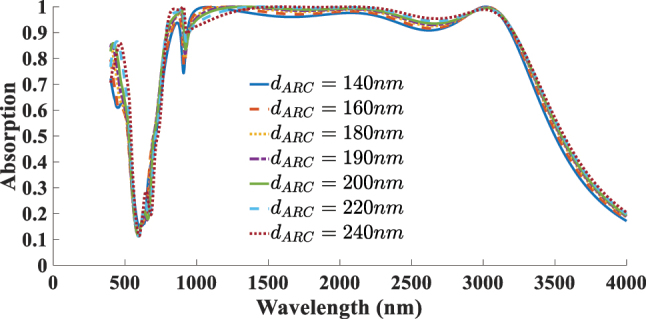
Dependent of absorption response to the thickness of the SiO_2_ (*d*_ARC_).

**Figure 14:**
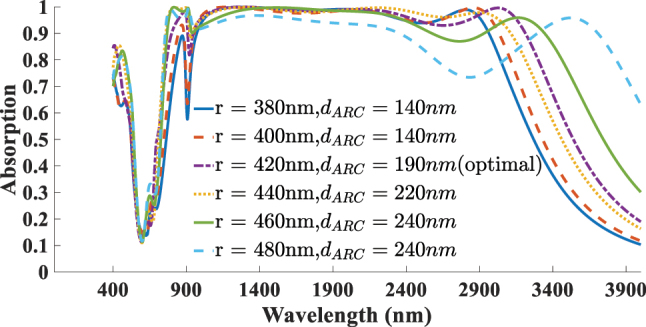
Optimal absorption spectra corresponding to the best ARC thickness for each selected resonator diameter, used to determine the optimal pair of (*r*, *d*_ARC_).

**Figure 15:**
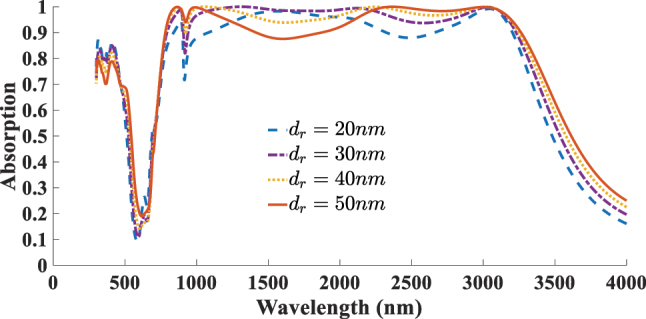
Dependent of absorption response to the thickness of the resonator (*d*_*r*_).

The next aspect addressed in this study is the impact of the absorber’s geometrical parameters on its absorption performance. As illustrated in Figure 12 the diameter of the resonator (*r*) is varied from 380 to 480 nm, with optimal absorption achieved *r* = 420 nm. Increasing the radius shifts the resonance toward longer wavelengths due to the larger effective cavity size, whereas reducing the radius weakens the coupling with the incident wave and narrows the absorption bandwidth. Figure 13 presents the effect of altering the thickness of the SiO_2_ ARC layer, varied within the range of 140–240 nm. The best performance is observed at *d*_ARC_ = 190 nm. For a more systematic optimization, we also investigated the simultaneous influence of the disk diameter and ARC thickness, which further confirms the robustness of our design. Figure 14 presents the optimal absorption spectra corresponding to the best ARC thickness for each selected resonator diameter. The highest absorption is achieved when both parameters are balanced to induce constructive interference and strong field confinement near the 2D material plane. This analysis identifies the optimal parameters as *r* = 420 nm and *d*_ARC_ = 190 nm. The next parameter investigated is the resonator layer thickness (*d*_*r*_). Figure 15 presents the absorption spectra for varying *d*_*r*_ from 20 to 50 nm, where the highest absorption and the broadest bandwidth are obtained at *d*_*r*_ = 30 nm.

**Figure 16:**
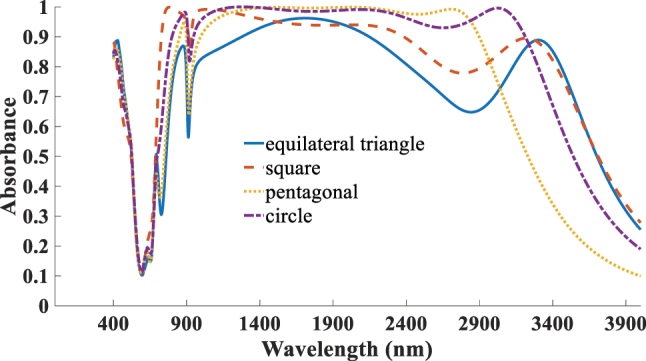
Simulated absorption spectra for various geometery of TiO_2_ resonator.

Finaly, to justify the choice of the disk geometry, we examined several resonator shapes-equilateral triangle, square, pentagonal, and circular, as shown in Figure 16. Among these, the circular disk exhibited the most stable and polarization-insensitive absorption response, as well as the broadest operational bandwidth. This advantage arises from its high rotational symmetry, which promotes uniform field confinement and minimizes localized scattering losses at sharp corners (commonly observed in polygonal designs). Moreover, circular disks provide a more continuous surface current distribution, leading to more efficient plasmonic coupling and improved impedance matching [40].

## 4 Comparison

Our investigations in the previous section led to the development of a record-level, exceptionally broadband borophene-based absorber with near-perfect absorption. The optimized key parameters of this structure are surface carrier density *n*_*s*_ = 1, resonator diameter *r* = 420 nm, ARC thickness *d*_ARC_ = 190 nm using SiO_2_, resonator thickness *d*_*r*_ = 30 nm and the borophene layer positioned on top of the resonators. This configuration operates optimally under transverse electric (TE) polarization. Furthermore, the same structure was examined with a two-dimensional graphene layer replacing borophene. The optimized parameters in this case were a Fermi energy of *E*_*f*_ = 0.9 eV, resonator diameter *r* = 420 nm, an ARC thickness *d*_ARC_ = 90 nm using SiO_2_, resonator thickness *d*_*r*_ = 30 nm and the graphene layer positioned on top of the resonators. Although both configurations demonstrated enhanced absorption performance, the borophene-based absorber exhibited significantly broader bandwidth and higher absorption levels compared to its graphene-based counterpart, highlighting the superior light confinement and absorption capabilities of borophene in the designed metastructure. Table 1 compares the absorption performance and bandwidth of the broadband absorbers proposed in this work with those recently reported. It proves record-level, exceptionally broadband behavior of our designed borophene based absorber.

**Table 1:** Comparing the absorption performance and bandwidth of the broadband absorbers proposed in this work with those of recently reported.

Reference	Structure	A > 90 % bandwidth	A > 80 % bandwidth
		Bandwidth	Bandwidth
[5]	Ti–SiO_2_–Ti–graphene	808–2,000 nm	758–2,000 nm
		1,192 nm	1,542 nm
[9]	Ag–graphene	988–1,494 nm	861–1,503 nm
		506 nm	642 nm
[10]	Au–SiO_2_–graphene–Ag, Au	488–1,371 nm	396–1,582 nm
		883 nm	1,186 nm
[30]	W–SiO_2_–graphene–W	373–1,967 nm	256–2,036 nm
		1,594 nm	1,780 nm
[41]	W–SiO_2_–graphene–W	847–1,036	829–1,051 nm
		189 nm	222 nm
[42]	Ag–alumina–graphene–Ag	1,531–1,582 nm	1,520–1,593 nm
		51 nm	73 nm
This work	Au–SiO_2_–Ti–graphene	923–2,108 nm	911–2,256 nm
		1,185 nm	1,345
This work	Au–SiO_2_–Ti–borophene	790–3,232 nm	760–3,306 nm
		**2,442** nm	**2,546** nm

The bold values indicate the exceptional, record-level bandwidths achieved by the proposed borophene-based absorber design.

## 5 Experimental feasibility

As a final section, it is important to address the experimental feasibility of the proposed design. The selected geometric and material parameters are within current experimental capabilities. Resonator dimensions (*r* = 420 nm, *d*_*r*_ = 30 nm) lie well above the resolution limits of standard electron-beam lithography (EBL) and are routinely fabricated by EBL followed by metal evaporation/sputtering and lift-off. These dimensions are also compatible with large-area replication via nanoimprint lithography. Anti-reflection coating (ARC) thicknesses in the 90–190 nm range can be deposited reproducibly using low-temperature atomic-layer deposition (ALD), thermal evaporation, sputtering, or by spin-coating polymer ARCs. A continuous Au ground plane and Ti resonators are straightforward to form by evaporation/sputtering. Integration of a 2D layer requires attention: borophene is typically synthesized epitaxially and is air-sensitive, so experimental realization will likely require controlled-environment transfer and encapsulation (for example h-BN capping) or *in situ* ARC transfer. To avoid damage during ARC deposition, non-destructive approaches such as low-temperature (<100 °C) ALD, spin-coating of polymer-based ARC materials, transfer of pre-formed ARC films, sacrificial buffer layers or remote (plasma-free) deposition can be employed. Electrostatic tuning to high Fermi levels (e.g., ≲0.9 eV) generally requires high-*κ* dielectrics and/or dual-gate or ionic-gating architectures. Chemical doping or ionic gating are alternative approaches to reach high carrier densities for borophene [26], [37], [38], [39], [43], [44], [45], [46], [47], [48], [49], [50], [51], [52], [53], [54], [55], [56]. Therefore, our proposed absorber is experimentally feasible, provided careful synthesis, encapsulation, and deposition strategies are used.

## 6 Conclusions

This work demonstrates the effectiveness of incorporating two-dimensional materials, specifically graphene and borophene, into a metal–insulator–metal (MIM) absorber structure to achieve ultra-broadband and high-efficiency light absorption. While both materials contribute to improved performance, borophene, when integrated with an optimized anti-reflection coating (ARC) layer, exhibits outstanding superiority, significantly surpassing graphene in terms of both absorption strength and spectral bandwidth. The proposed exceptionally broadband borophene-based absorber increases the absorption bandwidth from 73 % (ranging from 886 nm to 1,918 nm) to 121 % (from 790 nm to 3,232 nm) for absorptivity above 90 %, representing a remarkable 136 % enhancement over the base structure. For absorptivity above 80 %, the bandwidth extends to 125 % (from 760 nm to 3,306 nm), corresponding to a 125 % improvement. In comparison, the optimized graphene-based absorber achieves enhancements of 13 % and 12 % in bandwidth for absorptivity thresholds of 90 % and 80 %, respectively. Although graphene delivers narrower bandwidths, it still provides a substantial increase in absorption amplitude within its effective spectral range. Importantly, our analysis reveals the critical role of material placement in determining absorption performance. The borophene layer must be precisely located above the resonator to maintain high absorption efficiency, whereas graphene exhibits greater flexibility in its positioning without significant performance degradation. In addition, structural parameters such as the resonator’s geometry and dimension, and the ARC layer thickness, were shown to strongly influence the absorber’s efficiency, offering further opportunities for optimization. Our polarization analysis also confirms that the borophene-based structure performs better under transverse electric (TE) polarization, where the electric field is directed along the *x*-axis. Furthermore, we evaluated the influence of ARC material by replacing the conventional SiO_2_ layer with Si_3_N_4_. While this substitution led to minor shifts in the absorption spectrum, the overall performance remained below that achieved with the SiO_2_-based ARC layer. In summary, the findings emphasize the importance of 2D material selection and placement, polarization dependence, and geometrical tuning in maximizing absorber performance. These insights provide valuable design guidelines for next-generation photonic and optoelectronic devices, including broadband photodetectors, thermal camouflage, infrared imaging, and solar energy harvesting systems.
